# Enhanced Toxicity of Diol-Estered Diarrhetic Shellfish Toxins Across Trophic Levels: Evidence from *Caenorhabditis elegans* and *Mytilus galloprovincialis*

**DOI:** 10.3390/md23120459

**Published:** 2025-11-28

**Authors:** Caihong Chen, Haiyan Wu, Guanchao Zheng, Limin Lu, Zhijun Tan

**Affiliations:** 1 College of Food Science and Engineering, Ocean University of China, Qingdao 266404, China; 2 Key Laboratory of Testing and Evaluation for Aquatic Product Safety and Quality, Ministry of Agriculture and Rural Affairs, Yellow Sea Fisheries Research Institute, Chinese Academy of Fishery Sciences, Qingdao 266071, China; 3 State Key Laboratory of Mariculture Biobreeding and Sustainable Goods, Yellow Sea Fisheries Research Institute, Chinese Academy of Fishery Sciences, Qingdao 266071, China

**Keywords:** *Prorocentrum lima*, diol-estered diarrhetic shellfish toxins, toxicity assessment, oxidative stress, accumulation

## Abstract

*Prorocentrum lima* is a widely distributed and major source of diarrhetic shellfish toxins (DSTs); the ecological impact of diol-estered DSTs (eDSTs) compounds on benthic systems is still inadequate. In this study, the acute toxicity of eDSTs was evaluated in *Caenorhabditis elegans*, and their accumulation capacity and toxic effects were examined in *Mytilus galloprovincialis* for an ecological risk assessment. The results indicated that larvae 1 (L1) was more sensitive than larvae 4 (L4) of *C. elegans*, and the eDSTs in *P. lima* extract lysate were more toxic than the okadaic acid (OA) standard solution. The lowest LC_50_ values were 0.293 and 0.469 μg/mL for L1 and L4, respectively. The growth, productivity, and intestinal permeability of *C. elegans* were impaired, and the effect of *P. lima* extract lysate on *C. elegans* was greater than that of the OA standard solution. The total toxin concentration in the digestive gland of mussels reached 3230 μg/kg, with esterified DSTs accounting for 76.7–97.1% of total toxins and inducing marked oxidative stress. Diol-estered DSTs exert direct toxic effects, including oxidative damage and growth inhibition, while exhibiting a high accumulation potential. This study revealed the toxicity of eDSTs, necessitating a focused investigation to comprehensively assess their toxicological impact and ecological risks.

## 1. Introduction

Diarrhetic shellfish toxins (DSTs) are one of the most widely distributed and harmful marine biological toxins worldwide. They are primarily produced by benthic marine dinoflagellates such as *Prorocentrum lima* (*P. lima*). Benthic *P. lima* synthesizes high levels of diol-ester DSTs (eDSTs), such as okadaic acid (OA) and dinophysistoxins (DTXs), which are distributed among toxic algae and released into the surrounding seawater [1,2,3,4]. According to European Union (EU) standards, the maximum permissible level for DSTs is 160 μg/kg (OA equivalent) [5]. Bioaccumulation is initiated either through direct algal consumption or through the absorption of water-dissolved toxins excreted by microalgae [6]. Consequently, DSTs pose significant ecological threats, causing severe toxicity to various organisms, including microbial communities (affecting their diversity), zooplankton, and macrozoobenthic communities [7,8]. Notably, DSTs can exacerbate mortality and disease in marine organisms [9]. DSTs accumulate significantly in lipid-rich tissues of benthic marine organisms during harmful algal blooms (HABs), and their levels strongly correlate with *P. lima* cell abundance [10,11]. This bioaccumulation facilitates its widespread transfer through the food chain. The accumulation of DSTs can alter organism behavior and morphology, and in severe cases, lead to mass mortality [12]. Human consumption of seafood contaminated with OA and DTXs results in diarrhetic shellfish poisoning, characterized by acute gastrointestinal symptoms, including diarrhea, nausea, abdominal pain, and vomiting. Due to their significant ecological and public health impacts, DSTs and their producers are key targets of major international HAB research programs, such as GEOHAB and GlobalHAB [13].

DSTs primarily exert toxicity as potent inhibitors of protein phosphatases (PPs) such as PP2A and PP1 [14]. This results in broad-spectrum aquatic toxicity, including cytoskeletal damage and oxidative stress [15], and propagates multi-trophic adverse effects, including impaired growth, reduced reproduction, and compromised immune function across species [8]. Sub-chronic exposure induces cytotoxicity, immunotoxicity [16], and genotoxicity [17]. Long-term exposure to DSTs detrimentally affects critical physiological processes, which are manifested in feeding behavior and reproductive quantity in fish [8] and severe cellular and molecular damage in bivalve mollusks. The LD_50_ values of OA and DTX-1 for intraperitoneal injection [18] and oral administration [19] in mice were 150.4, 185.6, 487, and 760 μg/kg, respectively. This indicated that the toxicity of the individual DST components varied significantly. A similar pattern of differential toxicity was also observed in aquatic organisms. The LC_50_ values of OA and DTX-1 for zebrafish larvae exposed for 24 h were 10 and 7 μg/mL [20], and for medaka exposed for 96 h were 23.5 and 16.3 μg/mL [21]. Moreover, an increase in oxidative damage was observed in all cases. Therefore, the differences in toxicity might be primarily attributed to structural differences among the toxins.

The widespread distribution of DSTs and toxin-producing algae *P. lima* in Chinese coastal waters is well-documented [4]. The impacts on mariculture scale and production value are consequently elevated by increasing reported expansions [22]. Critically, *P. lima* strains from diverse geographic regions exhibit significantly high proportions (45–100%) of eDSTs derivatives at the single-cell level [23], with significant variations in toxin production and release observed across different locations [6]. These esterified derivatives dominate the toxin profiles within benthic food webs and often constitute > 70% of the total toxins detected [6]. Bivalve mollusks, such as oysters, can directly filter *P. lima* [24] and efficiently convert DSTs into fatty acid-esterified forms [25], with conversion rates reaching approximately 50% [26]. Notably, these esterified toxins accumulate significantly in higher trophic level organisms, including crabs, shrimps, and sea turtles [27], where they generally represent the predominant form (for instance, up to 70% as OA esters) [28]. Collectively, these findings demonstrate the significant bioaccumulation and potential biomagnification of esterified DST derivatives in the benthic food chain [29]. However, the mechanisms governing this transfer process (particularly in its esterified form) and its direct cytotoxic and broader ecological effects on benthic organisms remain poorly understood and represent a significant knowledge gap [30].

The higher toxicity of *P. lima* extract lysate compared to that of pure OA has been extensively proven, with studies consistently demonstrating strong cytotoxic effects in cellular systems [31,32,33,34]. Consequently, eDST derivatives may act as bioactive compounds, accumulating in bivalves, shrimp, crabs, and other aquatic organisms [35]. These complex exposure pathways pose a significant regulatory challenge due to uncertainties surrounding chronic risks. Overall, current evidence indicates that esterified forms can be more toxic, although research in this area remains limited. *Caenorhabditis elegans* (*C. elegans*) is extensively used in toxicological research due to its highly conserved biology, small size, and fast reproduction [36]. Moreover, its intestine is a direct target of environmental toxins, allowing for clear observation of damage and providing a robust model for investigating intestinal toxicity of DSTs [37]. In this study, the toxicity and accumulation character of eDSTs were investigated in *C. elegans* and *Mytilus galloprovincialis* were investigated: (i) to determine the effects of eDSTs on the ecotoxicological endpoints of *C. elegans*, including survival, growth, and reproduction; (ii) to determine the accumulation, transformation, and tissue-damaging effects in mussels. This study reveals eDSTs’ toxicity across trophic levels, thereby providing critical data to assess the ecological hazards and potential food chain transfer risks posed by *P. lima* blooms in natural environments.

## 2. Results

### 2.1. Acute Toxicity of DSTs to C. elegans

**Figure 1:**
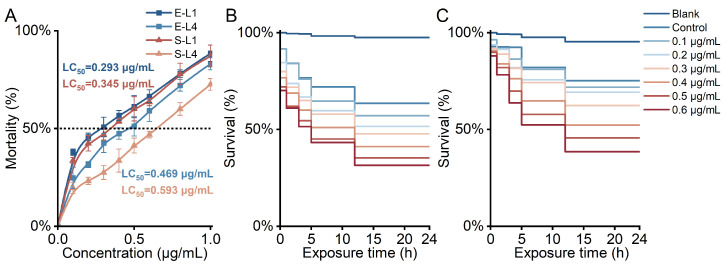
Acute toxicity comparison between *P. lima* extract lysate and OA standard solution of *C. elegans*. (**A**) Concentration–response mortality. (**B**) A 24 h survival rate of L1 larvae exposed to the *P. lima* extract lysate. (**C**) A 24 h survival rate of L4 larvae exposed to the *P. lima* extract lysate.

The concentration–response relationship of *P. lima* extract lysate and OA standard solution on the acute toxicity of *C. elegans* at stages larvae 1 (L1) and larvae 4 (L4) is illustrated in Figure 1A. The probit regression probability function was used for fitting, indicating an obvious agent effect relationship between the concentration and response. The fitted probability unit regression model is presented in Table S3. The LC_50_ of the *P. lima* extract lysate against nematodes at the L1 and L4 stages was calculated to be 0.293 and 0.469 μg/mL, respectively. Similarly, the LC_50_ of the OA standard solution against *C. elegans* at stages L1 and L4 was 0.345 and 0.593 μg/mL, respectively. The LC_50_ of the *P. lima* extract lysate was 15% and 21% lower than that of the OA standard solution at stages L1 and L4, respectively, suggesting a significantly higher toxicity than the OA standard solution.

The effects of DSTs on the 24 h survival rates of L1 and L4 larvae are presented in Figure 1B,C. Nematode survival decreased over time, with a negative correlation between the exposure concentration and survival rate. At a concentration approaching the respective LC_50,_ L1 larvae exposed to 0.3 μg/mL of *P. lima* extract lysate achieved an LT_50_ of 20.5 h with a final survival rate of 47.8%. L4 larvae exposed to 0.5 μg/mL of *P. lima* extract lysate achieved an LT_50_ of 19.5 h with a final survival rate of 45.6% (Table S4). At the highest tested dose (0.6 μg/mL), the L1 survival rate was 31.5%, whereas the L4 survival rate was 38.6%. Overall, these results demonstrate that L1 larvae exhibit higher susceptibility to *P. lima* extract lysate treatment than L4 larvae at equivalent exposure levels.

### 2.2. Effect of DSTs on Physiological Parameters in C. elegans

**Figure 2:**
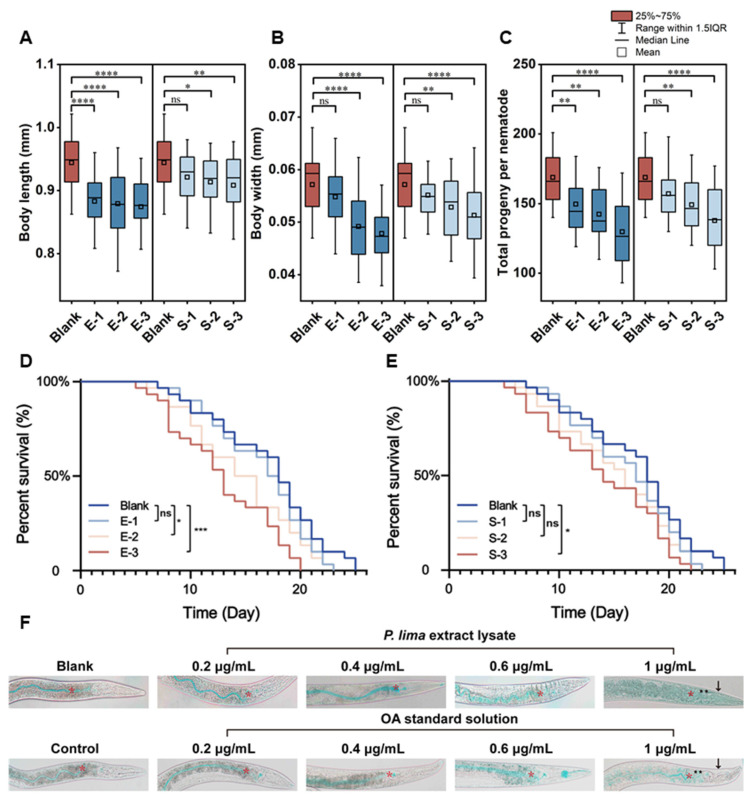
Graphical representation of the effects of DSTs on *C. elegans* physiological parameters. (**A**) Growth in body length (*n* = 30). (**B**) Growth of body width (*n* = 30). (**C**) Reproduction (*n* = 30). (**D**,**E**) Lifespan (*n* = 30). (**F**) Intestinal permeability, intestinal cells (**), and intestinal lumen (*) were labeled with asterisks, and the body lumen was labeled with arrowheads. * *p* < 0.05, ** *p* < 0.01, *** *p* < 0.001, **** *p* < 0.0001 were in comparison to Blank. Among them, E-1, E-2 and E-3 are 0.03, 0.06 and 0.15 μg/mL, respectively, and S-1, S-2 and S-3 are 0.03, 0.06 and 0.15 μg/mL, respectively.

The growth and reproduction of *C. elegans* were significantly inhibited after 48 h of DSTs exposure (Figure 2A–C). The blank group exhibited a body length of 0.94 ± 0.044 mm and a body width of 0.057 ± 0.006 mm. Conversely, exposure to *P. lima* extract lysate caused significant reductions in body length (6.5–7.4%; **** *p* < 0.0001) and width (2.4–3.8%; **** *p* < 0.0001). However, the OA standard solution group did not exhibit any significant effects (*p* > 0.05) on body dimensions at 0.03 μg/mL, with only a marginal 3.8% decrease in length at the highest concentration of 0.15 μg/mL (**** *p* < 0.0001). Notably, no concentration–response relationship was observed in growth parameters across treatments (*p* > 0.05) (Figure 2A,B).

Following a 48 h exposure, all nematodes stopped egg-laying on the fifth day after transferring to NGM plates. Compared with the blank group, DST-exposed nematodes demonstrated significantly reduced progeny production (*p* < 0.05; Figure 2C), except for the S-1 group (OA standard solution at 0.03 μg/mL; *p* > 0.05). Notably, *P. lima* extract lysate groups exhibited reductions of 11.4%, 15.6%, and 23.2%, whereas OA standard solution groups exhibited 6.5%, 11.3%, and 18.4% reductions, respectively.

Lifespan assessments revealed similar trends (Figure 2D,E). *P. lima* extract lysate exhibited significantly higher anti-reproductive activity compared to the OA standard solution (*p* < 0.05). The maximum lifespan of *P. lima* extract lysate and the OA standard solution was 20 and 22 days, respectively. DSTs significantly reduced lifespan by 2–5 days at 0.15 μg/mL (*p* < 0.05). Kaplan–Meier analysis revealed that the median survival time of the control group was 18 ± 1 days. The *P. lima* extract lysate group exhibited significant differences from the blank group at both 0.06 (*p* < 0.05) and 0.15 μg/mL (*p* < 0.001), whereas the OA standard solution group exhibited a significant difference only at 0.15 μg/mL (*p* < 0.05), suggesting a concentration–response relationship.

### 2.3. Effect of DSTs on Intestinal Permeability in C. elegans

DSTs caused acute toxicity by damaging intestinal permeability, as evidenced by the blue dye marking the intestinal lumen in all treatment groups (Figure 2F). Compared with the blank group, the intestinal lumen of the control group exhibited altered and distorted luminal morphology. The most significant observations were that the body became clear, dye translocation from the lumen to intestinal cells, and significant accumulation in the body cavity. The body becoming clear is primarily attributed to lipid depletion in the nematode. These effects were concentration–response and notably more pronounced in the *P. lima* extract lysate groups than in OA standard solution groups. The most severe effects were observed in the *P. lima* extract lysate group at a concentration of 1 μg/mL. After 12 h of exposure, the high-concentration groups (0.6 and 1 μg/mL) demonstrated increased mortality (>50%), with surviving nematodes stained and imaged. In conclusion, DSTs caused concentration–response intestinal barrier dysfunction with a significant increase in permeability at concentrations > 0.6 μg/mL.

### 2.4. Accumulation and Distribution of DSTs in Mussels

**Figure 3:**
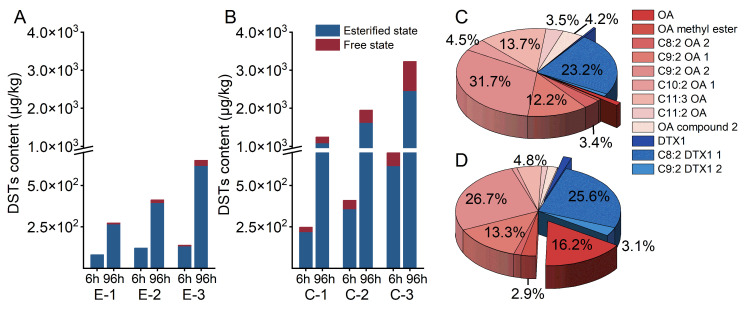
Accumulation of DSTs after 6 and 96 h of exposure and their esterified toxins after exposure for 96 h in mussels. (**A**,**C**) *P. lima* extract lysates. (**B**,**D**) Algal cell groups. Among them, E-1, E-2 and E-3 are 1, 2 and 4 μg/L, respectively, and C-1, C-2 and C-3 are 1 × 10^5^, 3 × 10^5^, and 6 × 10^5^ cells/L, respectively.

Mussels rapidly accumulate DSTs under both exposure methods, indicating significant positive correlations with both the exposure dose and duration. A mortality rate < 1% was observed in these experiments. After 6 h of exposure, the total DSTs content in the algal cell group (up to 700 μg/kg) was approximately five times higher than that in the *P. lima* extract lysate group (Figure 3A,B). This 5-fold difference persisted after 96 h, with the algal cell group accumulating up to 3230 μg/kg of total DSTs, whereas the *P. lima* extract lysate group accumulated up to 650 μg/kg. Notably, the toxin content increased progressively with exposure concentration, demonstrating a significant DST concentration–response relationship. After 96 h, esterified toxins dominated 76.7–97.1% of the total DSTs. HRMS further identified C9:2 OA2 and C8:2 DTX1-1 as the primary esterified forms, constituting a total ratio of 54.9% and 52.3% in the two groups, respectively (Figure 3C,D).

### 2.5. Effects of DSTs on Oxidative Activity Damage in Mussels

**Figure 4:**
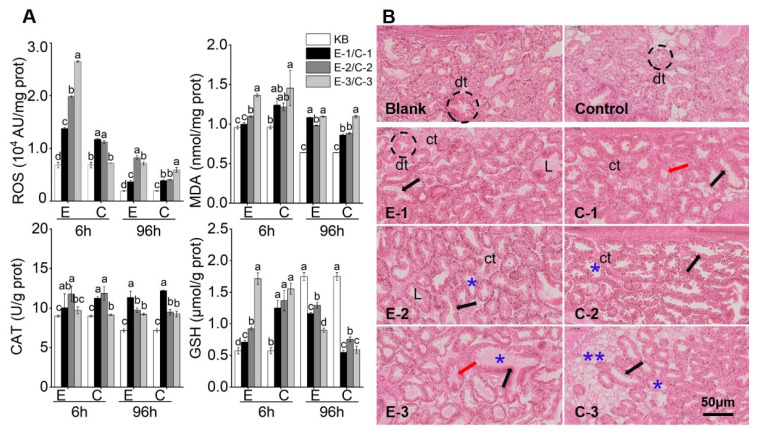
Response of DSTs to oxidative activity damage in mussels. (**A**) Antioxidant enzyme activity in mussel digestive glands. The letters above the bar chart represent significant differences among the groups. If the same letters were used between groups, the difference was not significant (*p* > 0.05). If there were no same letters, the difference was considered significant (*p* < 0.05). (**B**) Tissue sections of the mussel digestive glands. dt: Digestive tubules, ct: Connective tissue, L: Lumen of the tubules; *: Dilation of the small lumen, **: Degeneration of digestive cells, black arrow: Deformation of the digestive cavity, and red arrow: Infiltration of blood cells. Among them, E-1, E-2 and E-3 are 1, 2 and 4 μg/L, respectively, and C-1, C-2 and C-3 are 1 × 10^5^, 3 × 10^5^ and 6 × 10^5^ cells/L, respectively.

Figure 4A illustrates the antioxidant enzyme activities in the digestive glands of mussels following exposure to *P. lima* extract lysate or algal cell groups. Both treatments significantly altered ROS, GSH, MDA, and CAT levels (*p* < 0.05). Elevated ROS and MDA levels indicate significant oxidative stress, which is a major contributor to structural damage to the digestive gland. Lipid peroxidation (reflected by high ROS and MDA levels) damages cellular membranes, ultimately disrupting digestive tubule integrity. GSH activity was initially upregulated and was potentially linked to the observed decline in ROS levels from 6 to 96 h. Increased CAT activity reflects the response of an organism to toxin-induced oxidative stress by enhancing antioxidant defenses.

Histological analysis revealed that the digestive tubules in the blank group exhibited ciliated acidic epithelial cells with intact folds and a closed lumen (Figure 4B). After 96 h of exposure, both treatment groups suffered varying degrees of damage to the digestive glands of mussels, including distorted digestive cavities, atrophy and thinning of epithelial cells, and hemocyte infiltration. Notably, the *P. lima* extract lysate and algal cell groups exhibited severe cavity distortion accompanied by hemocyte infiltration, indicating that DSTs caused severe damage to the digestive glands of mussels. Furthermore, degeneration of the digestive tubules was observed in group C. The complexity of these effects increased with the exposure dose.

## 3. Discussion

Reports have demonstrated that blooms of *P. lima* can reach cell densities of 10^4^–10^6^ cells/L [1,3]. Our experimental concentrations (1 × 10^5^, 3 × 10^5^, and 6 × 10^5^ cells/L) were selected to encompass these environmentally relevant levels. In the standardized toxicological model *C. elegans*, the comparison between the *P. lima* extract lysate and the OA standard solution was designed to isolate and quantify the enhanced intrinsic toxicity of eDSTs, whereas in the ecologically relevant filter-feeder *M. galloprovincialis*, the comparison between the *P. lima* extract lysate and algal cells aimed to mimic natural exposure routes and evaluate the critical role of algal cells as toxin vectors in bioaccumulation. Toxicity assessment revealed that eDSTs induced higher intestinal permeability and toxicity in nematodes, along with increased bioaccumulation potential and tissue damage in mussels. These findings reveal enhanced toxicity of diol-estered diarrhetic shellfish toxins across trophic levels.

### 3.1. Acute Toxicity and Difference Assessment

The acute toxicity of OA to *C. elegans* had not been reported prior to this study. Our findings address this knowledge gap by demonstrating that L1 larvae are more sensitive to OA than L4 larvae, a result consistent with patterns observed in other species where earlier life stages often show increased susceptibility. For instance, in *Artemia*, the 24 h LC_50_ for Instar I larvae (0.134 μg/mL) is significantly lower than that for adults (0.186 μg/mL) [8,38]. *Artemia* was more sensitive to OA than *C. elegans* (LC_50_: 0.345–0.593 μg/mL). Different developmental stages exhibit diverse sensitivities, mainly owing to detoxification systems and metabolic rates [39]. In contrast, vertebrate fish such as zebrafish (LC_50_: 7–10 μg/mL) and Japanese medaka (LC_50_: 23.5 μg/mL) exhibit considerably higher LC_50_ values [18,20], which are approximately 12–40 times greater than those of *C. elegans*. This establishes a clear sensitivity gradient: *Artemia* > *C. elegans* > fish, suggesting that larger and more complex organisms may possess higher tolerance to OA in natural environments.

Furthermore, we observed that eDSTs in the *P. lima* extract lysate demonstrated higher toxicity than the OA standard solution. Their LC_50_ values were 0.293 (L1) and 0.469 μg/mL (L4), respectively, which were 21% lower than those of OA, confirming that the toxic effect of eDSTs was amplified. Sea bass fed live *P. lima* cells had 90% mortality, whereas those consuming *P. lima*-contaminated Artemia exhibited 75% mortality, implying the superior toxicity of intact algal cells [40]. Another study demonstrated that medaka embryos exposed to the extract lysate required a 3.4-fold lower equivalent concentration of OA (0.22 μg/mL) than OA (0.75 μg/mL) to achieve 100% mortality [31], indicating that the extract contained additional bioactive compounds that increased the toxicity. Beyond lethality, the *P. lima* extract lysate resulted in a greater increase in intestinal permeability in *C. elegans* than the OA standard solution, exacerbating barrier dysfunction, which may be correlated with the higher lipophilicity of eDSTs, thereby promoting intestinal barrier penetration and stronger PP2A inhibition.

In this study, OA and its esterified derivatives severely impaired the growth and reproductive capacity of *C. elegans*. Comparatively, eDSTs exhibited higher toxicity, further reducing L1 larval survival and accelerating aging. This enhanced toxicity effect has been demonstrated in numerous models. For instance, medaka larvae simultaneously exhibit abnormal changes in oxidative parameters and persistent morphological damage [21], whereas *P. lima* extract lysate induces significant oxidative stress in zebrafish [20]. As a potent phosphatase inhibitor, OA dysregulates actin filament organization via phosphoproteomic alterations that affect cytoskeletal proteins [41]. This study highlights explicitly OA-induced intestinal damage in *C. elegans*, characterized by increased permeability, and verifies the established toxicity endpoints in nematode models [42,43]. This pathology mirrors mammalian diarrhetic shellfish poisoning, in which barrier dysfunction causes diarrhea. Crucially, eDSTs demonstrate increased toxicity because of their greater lipophilicity, which facilitates intestinal barrier penetration. Therefore, the enhanced toxicity of *P. lima* extract lysate is primarily driven by eDSTs via phosphatase inhibition [44,45], although potential contributions from other unidentified bioactive compounds may also be present.

### 3.2. Bioaccumulation and Ecological Risk

Bivalves tolerate DSTs through coordinated physiological adaptations, despite significant toxin accumulation. Field investigations have reported DST concentrations of 37.3 μg/kg in mariculture zones of the Chinese Yellow Sea and peak levels of 107 μg/kg in Brazilian coastal mussels [35,46]. In our experiments, mussels exposed to *P. lima* accumulated more than five times more toxins in their digestive glands (up to 3230 μg/kg) than in aqueous medium exposure. Mussel mortality was zero despite exposure concentrations exceeding those of the field survey, indicating that mussels have a tolerance ability [47]. Esterified toxins, which accounted for 76.7–97.1% of the total DSTs, dominated the profile and exhibited a composition similar to the *P. lima* extract lysate. This suggests that their accumulation within 96 h likely involved both direct uptake from the algal source and subsequent tissue transfer. Therefore, the food chain mainly spreads in the esterified form. However, current studies have primarily focused on total DSTs, which may lead to a significant underestimation of the actual ecological and health risks due to the higher toxicity and bioaccumulation potential of the esterified forms.

The antioxidant enzyme system plays a significant role in defense against oxidative stress. In this study, acute exposure to DSTs significantly increased the activity of antioxidant enzymes in mussels, including MDA and CAT, indicating a toxin-induced oxidative damage pathway. Furthermore, ROS-mediated membrane destruction enhances the uptake and accumulation of lipophilic toxins [48]. This is comparable to the upregulation of GST-ω and GPx genes in oysters and clams exposed to *P. lima*, which counteract oxidative stress as a defense against DSTs [49]. Although this can reduce the impact of DSTs, bivalves still suffer certain damage, such as changes in tissue morphology that may intuitively reflect their physiological changes [50]. In this study, the digestive glands of mussels indicated degeneration of digestive cells, deformation of the digestive cavity, and infiltration of blood cells. Similarly, studies have demonstrated that exposure to *P. lima* increases blood cell infiltration in the digestive organs of mussels (47%) [50]. Oysters exposed to *P. lima* exhibit abnormal structural changes in their digestive glands, including partial degeneration of the epithelium, thinning of the epithelium, and blood cell infiltration inside the tubules [47]. In conclusion, tissue damage is a manifestation of an organism’s immune defense, together with oxidative stress markers (ROS, CAT, and MDA), constituting physiological responses to toxin exposure. However, the ultimate manifestation of digestive gland injury indicates that the intrinsic repair capacity has been surpassed by the toxin-induced damage.

Research on the toxicity of eDSTs remains limited; however, accumulating evidence confirms their enhanced toxicity relative to free forms. In vivo evidence from mouse intraperitoneal injection studies confirms that eDSTs exhibit enhanced toxicity compared to their free forms [2]. At the cellular level, all diol-ester toxins isolated from *P. lima* showed cytotoxic effects, with the most potent ones exhibiting activity comparable to that of OA [34]. The underlying mechanism is attributed to the ability of these esters to be effectively taken up by cells and hydrolyzed intracellularly, releasing the active toxins [51]. Furthermore, the toxic potency of different OA equivalents displayed a consistent trend in both PP2A inhibition and cytotoxicity assays, and correlated more closely with oral toxicity in mice than with intraperitoneal toxicity [52]. These findings collectively suggest that PP2A inhibition serves as a robust and predictive indicator for assessing the toxicity of various esterified toxins.

Bivalves primarily detoxify DSTs through esterification, converting toxins such as OA into fatty acid esters (for instance, DTX-3 and DTX-4) [53]. For instance, approximately 50% of OA is bioconverted into mussels [54], whereas oysters exhibit a higher conversion efficiency of 83–93% [55]. However, the biotransformation of DSTs is not limited to esterification. Progressive hydrolysis further modifies these toxins. Non-toxic sulfated diesters (DTX-4) are rapidly cleaved into lipophilic diol-esters, which slowly release the free state [56]. This multistep process explains the coexistence of diverse toxin forms in cells [51], paradoxically revealing their potential for perpetuation in the food chain.

Importantly, hydrolysis alters the polarity of the toxin into neutral diol-esters, increasing membrane permeability and promoting bioaccumulation [56]. However, knowledge regarding the biomagnification of eDSTs across higher trophic levels and their long-term ecological impacts remains particularly limited. Recent studies have emphasized the increasing bloom risk of *P. lima* (a major OA producer) in Chinese coastal waters [22], underscoring the urgency to assess toxin accumulation in benthic organisms and their predators. Therefore, a comprehensive assessment framework is needed to evaluate the transmission potential of eDSTs through food webs and their associated ecological risks.

## 4. Materials and Methods

### 4.1. Reagents and Materials

Reagents: Methanol (chromatographic grade) and acetonitrile (mass spectrometry grade) were procured from Merck (Rahway, NJ, USA). Chromatographic grade formic acid and ammonium formate were obtained from Fluka (Buchs, Switzerland). Hydrochloric acid and sodium hydroxide were of reagent grade. Water (18 MΩ cm) was deionized and passed through a Milli-Q water purification system (Millipore, Billerica, MA, USA). Certified reference materials for OA and DTX1 were obtained from the National Research Council of Canada (Halifax, NS, Canada). Physiological saline solution (0.9%, sterile) was obtained from Servicebio (Wuhan, China). A 1× phosphate-buffered saline (0.01 M, pH 7.2–7.4) was procured from Solarbio (Beijing, China). Nematode growth medium (NGM) was obtained from Topbio (Yantai, China). Levamisole hydrochloride (≥99%) was obtained from Shanghai Aladdin Biochemical Technology Co., Ltd (Shanghai, China).

Solution preparation: K solution: 0.051 M NaCl and 0.032 M KCl were dissolved in water. M9 buffer: 0.085 M NaCl, 0.001 M MgSO_4_, 0.022 M KH_2_PO_4_, and 0.022 M Na_2_HPO_4_·12H_2_O were dissolved in water. Nematode bleaching solution: 4 M NaOH: 0.1% NaClO: H_2_O = 1:2:7 (*v*:*v*:*v*). 5% erioglaucine disodium (Shitou, Shanghai, China): 5 g was dissolved in 100 mL of water.

### 4.2. Material Preparation

#### 4.2.1. Animals

The wild-type *C. elegans* Bristol N2 strain was obtained from the Caenorhabditis Genetics Center (CGC). The worms were cultured at 20 °C on NGM plates seeded with *Escherichia coli* OP50 (*E. coli* OP50). NGM was poured into disposable Petri dishes, air-dried and inoculated with *E. coli* OP50 on clean bench until bacterial lawn was formed.

Mussel larvae (*Mytilus galloprovincialis*, 30 ± 10 mm anterior–posterior shell length) were procured from Qingdao, Shandong Province, China. Appropriate individuals were selected and transported to the laboratory for cultivation at low temperature. After being transported to the laboratory, the mussels were cleaned with seawater (obtained from the natural seawater of Huiquan Bay, Qingdao, China) to remove any sediment or algae that adhered to the surface. Subsequently, they were placed in a culture box containing clean seawater for acclimatization and adaptation. During this period, an oxygen pump was used for continuous inflation with a 12 h light–dark cycle. The water temperature was maintained at 16 ± 3 °C, and the water in the tank was changed 1 h before each feeding. A total of 800 mussels were acclimatized to laboratory conditions for a week and fed with microalgae (*Chlorella vulgaris*). Samples were stored at −80 °C until analysis.

#### 4.2.2. Algal Cell Culture and Extraction Lysate Preparation

The SHG strain of *P. lima,* isolated from the Bohai China Sea (40°0′36″ N, 119°54′36″ E), was provided by the Research Center for Harmful Algae and Marine Biology of Jinan University (Guangzhou, China). The culture conditions were 22 ± 1 °C, with a 12 h light–dark cycle in a light incubator (MGC-450BP-2, Yiheng, Shanghai, China). The algal strains were inoculated into 225 cm^2^ cell culture flasks for adherent growth. Filtered seawater (0.45 μm) was boiled, supplemented with f/2 medium, and used for algal culture at room temperature.

*P. lima* extract lysate: During the stationary phase, cell density was 3000 cells/mL, and 200 mL of algal culture was aliquoted into four 50 mL centrifuge tubes and centrifuged at 1000× *g* for 10 min. The pellet was resuspended in 10 mL of 80% methanol–water and sonicated on ice using a 6 mm diameter tapered microtip probe (Scientz-IID Ultrasonic Cell Crusher, Scientz Biotechnology, Ningbo, China) at a 20% duty cycle and 130 W for 10 min. After sonication, the lysate was centrifuged at 8000× *g* for 5 min, and the supernatant was collected as the crude toxin extract. The extract was concentrated using a nitrogen stream (N-EVAP 112 Nitrogen Evaporators, River Road West Berlin, MA, USA), reconstituted in 20 mL of 50% methanol–water, and stored at −80 °C. Using the methods of Section 4.4.3, the total toxin concentration and the proportion of eDSTs of the *P. lima* extract lysate were 6.207 ng/mL and 55.8%, respectively (Tables S1 and S2).

### 4.3. Toxicity Assessment of DSTs to C. elegans

#### 4.3.1. Synchronization

After stabilizing the worm population, a synchronized bacterial community was established. To prepare L1 worms, pregnant adults were treated with a bleaching solution to obtain eggs. Briefly, a population of *C. elegans* was cultivated on 65 mm NGM plates until abundant egg deposition occurred. When there were many eggs on the plate, all worms and eggs were collected in a 15 mL centrifuge tube at 1000× *g* for 1 min and treated with a bleaching solution to isolate eggs at room temperature for 6 min. The samples were then centrifuged at 3000× *g* for 5 min. The eggs were washed thrice with M9 buffer, plated on *E. coli* OP50-free NGM plates, and incubated for 12–18 h to obtain synchronized L1 larvae. L1 larvae were collected and transferred to NGM plates seeded with *E. coli* OP50 and cultured for 48 h to reach the L4 stage for toxicity assessment. Nematodes that failed to respond to touch stimuli were considered dead.

#### 4.3.2. Acute Toxicity

To compare 24 h acute toxicity between synchronized L1 and L4 stage *C. elegans*, larvae were exposed to *P. lima* extract lysate (E) and OA standard solution (S). Toxin dilutions (0, 0.1, 0.2, 0.3, 0.4, 0.5, 0.6, 0.8, and 1.0 μg/mL) were diluted with sterilized K solution in 96-well plates in triplicate for each concentration. Each concentration was tested in triplicate wells containing 200 μL of the solution and at least 50 nematodes. Nematode mortality was assessed at 0, 1, 3, 5, 12, and 24 h after toxin exposure.

#### 4.3.3. Physiological Parameters

For physiological parameter evaluation, synchronized L1 larvae (30 worms/plate) were exposed to 30 mm NGM plates seeded with *E. coli* OP50. Test groups included *P. lima* extract lysate (E-1, E-2, and E-3) and OA standard solution (S-1, S-2, and S-3) at 0.03, 0.06, and 0.15 μg/mL, with K-medium and 5% methanol solution as blank and solvent controls, respectively. All conditions were tested in six replicates. After 48 h of exposure (reaching the L4 stage), the larvae were transferred to new NGM plates for assessment.

For growth determination, L4 larvae were cultured for 24 h until reaching the adult stage. The nematodes were paralyzed with levamisole (20 mM), photographed under a microscope, and analyzed using ImageJ 1.8 software to determine the body length and width of *C. elegans*.

For lifespan determination, dead nematodes were counted every 24 h, and surviving nematodes were transferred to a fresh NGM plate containing *E. coli* OP50. This process was repeated until all nematodes died, and the time was recorded.

For brooding size determination, the number of eggs per plate was counted, and nematodes were transferred to a fresh NGM plate every 24 h until egg laying stopped. The average daily egg-laying quantity in each group was recorded.

#### 4.3.4. Intestinal Permeability

Intestinal permeability was assessed by staining *C. elegans* with erioglaucine disodium. L4 larval nematodes were transferred to centrifuge tubes and exposed to 500 μL of toxin solutions (*P. lima* extract lysate or OA standard solution at 0.2, 0.4, 0.6, or 1.0 μg/mL). After 12 h of exposure, the toxin solutions were discarded and replaced with 500 μL of *E. coli* OP50 and 500 μL of erioglaucine disodium. Nematodes were washed with M9 buffer to eliminate any residual dye after 3 h of incubation at room temperature under light-protected conditions. A bright-field microscope (Nikon Eclipse 80i, Nikon Instruments Inc., California, USA) was used to image the nematode intestinal tract under optimal illumination. A minimum of 30 nematodes were examined for each experimental group.

### 4.4. Toxicity and Accumulation of DSTs in Mytilus galloprovincialis

#### 4.4.1. Exposure Experiment

**Figure 5:**
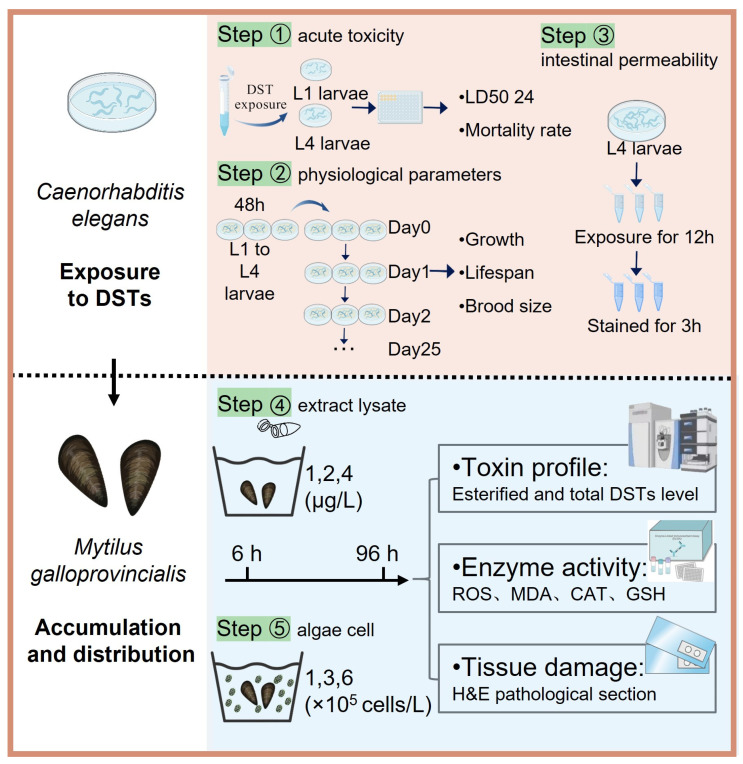
Nematode toxicity and mussel experiment diagram.

After seven days of acclimatization, mussels with good activity and uniform size were randomly selected for the experiment. The acute exposure experiment was conducted in 46 cm × 33 cm × 15 cm culture tanks. Each group used 2 L of seawater. Figure 5 illustrates the exposure groups, with 100 mussels in each group. All groups received supplemental feeding of *Chlorella vulgaris* (1 × 10^7^ cells/L) concurrent with toxin exposure. Seawater was renewed daily, mussel survival was monitored throughout the exposure period, and samples were collected at 6 and 96 h of exposure.

The control group included the blank and 5% methanol solvent control groups, whereas the experimental group included *P. lima* extract lysate (E) and algal cell groups (C). The exposure concentrations were determined as follows: For the algal cell group, based on “Red Tide Disaster Emergency Response Plan”, the reference density of *P. lima* was 5 × 10^5^ cells/L, and three exposure concentrations of 1 × 10^5^, 3 × 10^5^, and 6 × 10^5^ cells/L were set as C-1, C-2, and C-3, respectively. For the *P. lima* extract lysate group, based on the reference density and the single-cell toxin production of *P. lima* in the laboratory, approximately 6.5 pg/cell, three exposure concentrations of 1, 2, and 4 μg/L were established as E-1, E-2, and E-3 groups, respectively.

#### 4.4.2. Toxin Extraction

The toxin content in mussels was determined by collecting digestive glands from 50 mussels per group after 6 and 96 h of exposure (15 mussels per sample), with three replicate samples prepared. The samples were stored at −80 °C for analysis.

Samples were homogenized, and 1.00 ± 0.01 g of each sample was transferred to 5 mL centrifuge tubes. Methanol (2 mL) was added and mixed for 1 min, centrifuged at 6000× *g* for 10 min, and the supernatant was transferred to another 10 mL centrifuge tube. The precipitate was extracted in duplicate with 2 mL of methanol, and the volume of the pooled supernatant was adjusted to 5 mL. A 1 mL portion of the extract was filtered through a 0.22 μm nylon syringe filter (Agela Technologies, Tianjin, China) into a chromatography vial for instrumental analysis. This sample vial was identified as a free and esterified DSTs.

For the extraction of the total toxin content, 1 mL of the toxin extract was transferred to a 1.5 mL centrifuge tube containing 125 μL of NaOH solution (2.5 mol/L). The mixture was vortexed for 30 s, sealed, and incubated at 76 °C for 50 min in a water bath. After cooling to room temperature, 125 μL HCl (2.5 mol/L) was added for neutralization. The solution was thoroughly mixed, filtered through a 0.22 μm nylon membrane into a chromatography vial, and analyzed for toxin quantification. The total DSTs were identified in this sample vial.

#### 4.4.3. Analysis of DSTs by LC-HRMS/MS

An Ultimate 3000 High-performance liquid chromatography (HPLC) system (Thermo Fisher Scientific, MA, USA) equipped with a binary pump and autosampler was used for chromatographic separation. Analysis was performed using a Kinetex C8 column (150 mm × 2.1 mm, 2.6 µm; Phenomenex, Torrance, CA, USA) maintained at 40 °C. The mobile phases consisted of (A) water and (B) acetonitrile/water (95:5, *v*/*v*), both containing 0.2% formic acid and 2 mM ammonium acetate. Gradient elution was performed at a flow rate of 0.35 mL/min, starting with 20% B and increasing to 90% B over 5 min. The total run time was 15 min per injection (10 μL of the methanol extract).

For high-resolution mass spectrometry (HRMS) analysis, the HPLC system was coupled online to a Q Exactive hybrid quadrupole-Orbitrap mass spectrometer (Thermo Fisher Scientific, San Jose, CA, USA) using a heated electrospray ionization source (HESI-II). HESI-II parameters were set as follows: spray voltage, 3.5 kV; capillary temperature, 320 °C; heater temperature, 350 °C; sheath gas, 40 arb; auxiliary gas, 10 arb. Data acquisition was carried out in the positive ionization mode using a full MS/dd-MS^2^ strategy. Full MS scans were acquired at a resolution of 70,000 FWHM (*m*/*z* range 600–1500), and dd-MS^2^ scans were triggered at a resolution of 17,500 FWHM using stepped normalized collision energy (10/30 and 20/40 eV). Other MS^2^ settings included an AGC target of 5 × 10^5^ and a maximum injection time of 50 ms.

#### 4.4.4. Analysis of Oxidation Activity

Determination of oxidation-related enzyme activity: After 96 h of exposure, 10 mussels were randomly selected from each treatment group, and the digestive glands were isolated in triplicate. All samples were immediately frozen in liquid nitrogen and stored at −80 °C until further analysis. The manufacturer’s instructions were followed when using reactive oxygen species (ROS), Catalase (CAT), Malondialdehyde (MDA), glutathione (GSH) kits (Nanjing Jiancheng Bioengineering Institute, Nanjing, China), and Microplate Reader (iMark168-1130, Bio-Rad, Hercules, CA, USA).

Histopathological analyses: After 96 h of exposure, the digestive glands (*n* = 3) were fixed in a general-purpose tissue fixative (Servicebio, Wuhan, China) for 48 h at room temperature. The tissues were subsequently embedded in paraffin, sectioned at 4 μm thickness, and stained with hematoxylin and eosin (Meimian Biology, Yancheng, China). Subsequently, images of the stained digestive glands were analyzed using a MIDI scanner (3D-Histech, Budapest, Hungary) equipped with a case viewer software. Slide Viewer software (version 2.6) was used for slice observation.

### 4.5. Data Analysis

Statistical analysis was conducted using Statistical Package for the Social Sciences software (SPSS version 27.0). One-way analysis of variance was used to compare significant differences among the different test groups. The Shapiro–Wilk test was used to assess the normality of the data, and the Levene test was used to assess the homogeneity of variance before performing the parameter tests (*p* > 0.05). Least significant difference (LSD) and Duncan’s tests were used for post hoc multiple comparisons. Life span analyses were performed using Kaplan–Meier survival analyses with the log-rank test, and the statistical significance was set at *p* < 0.05, *p* < 0.01, and *p* < 0.001. The experiment was performed in triplicate, and the results are expressed as the mean ± standard deviation. The drawing was created using Origin 2024, ImageJ (version 1.8), and Adobe Illustrator 2023. The median lethal concentration and median lethal time (LC_50_ and LT_50_) were calculated using the probit regression function in SPSS [57].

## 5. Conclusions

This study established eDSTs as ecological keystone toxins by integrating nematode toxicity mechanisms, toxin accumulation and transformation in mussels, and the ecosystem transport model. The main findings include the following: (i) the acute toxicity of eDSTs to nematodes is significantly higher than that of free DSTs, including growth lifespan and intestinal permeability. (ii) The high accumulation of DSTs in mussel internal organs reached 3230 μg/kg, and the esterified form of toxins accounted for >76.7%. The exposure of algal cells was five times higher than that of the *P. lima* extract lysate. (iii) DSTs exert toxicity by influencing intestinal damage in nematodes, and esterification in bivalves and benthic food webs necessitates urgent monitoring of esterified derivatives in seafood. Our research revealed the toxicity of the esterified state and its differences among various organisms and provided theoretical and data support for ecological threats.

## Data Availability

The data presented in this study are available upon request from the corresponding author.

## Supplementary Materials

The following supporting information can be downloaded at: https://www.mdpi.com/article/10.3390/md23120459/s1. Table S1: The content of DSTs toxin in *P. lima* extract lysate in the experiment; Table S2. The proportion of each component of diol-estered DSTs in *P. lima* extract lysate; Table S3. Probit model fitting and LC_50_ (*n* ≥ 50); Table S4. Probit model fitting and LT_50_ (*n* ≥ 50).
