# Supplementary material for: Enhanced Toxicity of Diol-Estered Diarrhetic Shellfish Toxins Across Trophic Levels: Evidence from Caenorhabditis elegans and Mytilus galloprovincialis
Source: Mar Drugs. 2025 Nov 28;23(12):459. doi: 10.3390/md23120459 (PMC12735357; doi:10.3390/md23120459)
Supplement: Supplementary file 1 [file marinedrugs-23-00459-s001.zip › marinedrugs-3982514-supplementary.pdf]

Enhanced toxicity of diol-estered diarrhetic shellfish toxins across trophic levels: evidence from *Caenorhabditis elegans* and *Mytilus galloprovincialis*

Caihong Chen[a,b], Haiyan Wu[b], Guanchao Zheng[b], Limin Lu[a,b], Zhijun Tan[b,c]*
[a]College of Food Science and Engineering, Ocean University of China, Qingdao, 266404, China
[b]Key Laboratory of Testing and Evaluation for Aquatic Product Safety and Quality, Ministry of Agriculture and Rural Affairs, Yellow Sea Fisheries Research Institute, Chinese Academy of Fishery Sciences, Qingdao, 266071, China
[c]State Key Laboratory of Mariculture Biobreeding and Sustainable Goods, Yellow Sea Fisheries Research Institute, Chinese Academy of Fishery Sciences, Qingdao, 266071, China
* Corresponding author: Zhijun Tan
E-Mail: tanzj@ysfri.ac.cn
Phone: (86)0532-85836348; (86)18561605185
Fax: (86)0532-85825917

Table S1. The content of DSTs toxin in *P. lima* extract lysate in the experiment.

P. lima strain	Toxin types	Concentration (ng/mL) or Percentage (%)
SHG	Total DSTs	6.207
	Diol-estered DSTs	55.8%

Table S2. The proportion of each component of diol-estered DSTs in *P. lima* extract lysate.

Toxin	Toxin profile	Percentage (%)
OA	OA me	13.8%
	C8:2 OA1	7.3%
	C9:3 OA	17.0%
	C9:2 OA1	47.3%
	C10:2 OA1	5.3%
	C11:2 OA	0.3%
	OA components2	0.3%
DTX1	DTX1 me	0.2%
	C9:2 DTX1 1	8.5%

Table S3. Probit model fitting and LC_{50} (n≥50).

Group	Toxic solution	Probit Model	LD_{50}/（μg/mL）	95% confidence interval /(μg/mL)	P-value
E-L1	*P. lima* extract lysate	y=-0.467+0.002x	0.293	0.248～0.361	＜0.001
E-L4		y=-0.893+0.002x	0.469	0.418～0.535	＜0.001
S-L1	OA standard solution	y=-0.697+0.002x	0.345	0.287～0.402	＜0.001
S-L4		y=-1.022+0.002x	0.553	0.498～0.608	＜0.001

Table S4. Probit model fitting and LT_{50} (n≥50).

Group	Toxic solution	Probit Model	LT_{50}/（h）	95% confidence interval /(h)	P-value

E-L1	*P. lima* extract lysate	y=-0.683+0.033x	20.5	17.8～24.2	＜0.001
E-L4		y=-0.98+0.05x	19.5	16.0～25.3	＜0.001
